# Ecology and genetics affect relative invasion success of two *Echium* species in southern Australia

**DOI:** 10.1038/srep42792

**Published:** 2017-02-17

**Authors:** Xiaocheng Zhu, Paul A. Weston, Dominik Skoneczny, David Gopurenko, Lucie Meyer, Brendan J. Lepschi, Ragan M. Callaway, Geoff M. Gurr, Leslie A. Weston

**Affiliations:** 1 Graham Centre for Agricultural Innovation (Charles Sturt University and NSW Department of Primary Industries), Charles Sturt University, Wagga Wagga, 2678, Australia; 2 NSW Department of Primary Industries, Wagga Wagga Agricultural Institute, Wagga Wagga, 2650, Australia; 3 Australian National Herbarium, Centre for Australian National Biodiversity Research, Canberra, 2601, Australia; 4 Division of Biological Sciences, University of Montana, Missoula, 59812, USA; 5 Institute of Applied Ecology, Fujian Agriculture & Forestry University, Fuzhou 350002, China

## Abstract

*Echium plantagineum* and *E. vulgare* are congeneric exotics first introduced to Australia in the early 1800 s. There, *E. plantagineum* is now highly invasive, whereas *E. vulgare* has a limited distribution. Studies were conducted to evaluate distribution, ecology, genetics and secondary chemistry to shed light on factors associated with their respective invasive success. When sampled across geographically diverse locales, *E. plantagineum* was widespread and exhibited a small genome size (1 C = 0.34 pg), an annual life cycle, and greater genetic diversity as assessed by DNA sequence analysis. It was found frequently in areas with temperature extremes and low rainfall. In contrast, *E. vulgare* exhibited a larger genome size (1 C = 0.43 pg), a perennial lifecycle, less chloroplast genetic diversity, and occurred in areas with lower temperatures and higher rainfall. Twelve chloroplast haplotypes of *E. plantagineum* were evident and incidence aligned well with reported historical introduction events. In contrast, *E. vulgare* exhibited two haplotypes and was found only sporadically at higher elevations. *Echium plantagineum* possessed significantly higher levels of numerous pyrrolizidine alkaloids involved in plant defence. We conclude that elevated genetic diversity, tolerance to environmental stress and capacity for producing defensive secondary metabolites have contributed to the successful invasion of *E. plantagineum* in Australia.

Introduced species are of global concern in terms of their inherent economic and environmental costs, with annual losses of USD $1.4 trillion associated with biological invaders around the world^1^. Australia has endured the ravages of numerous noxious invaders [e.g. prickly pear cactus (*Opuntia stricta* (Haw.) Haw), cane toad (*Rhinella marina* L.), carp (*Cyprinus carpio* L.) and European rabbit (*Oryctolagus cuniculus* L.)], many of which were intentionally introduced from overseas. In terms of invasive Australian plants, agricultural costs of weed management alone are reported to exceed $4 billion annually and all of the most noxious weeds are non-indigenous^2^.

Successful plant invaders often rapidly adapt to novel ecosystems^3^. This can be achieved through rapid occupation of an empty niche^4^, “evolution of increased competitive ability”^5^, increasing colonizing ability^6^, production of large amounts of viable and long-lasting seeds^7^, a lack of enemies in the non-native range^8^, synthesis of allelochemicals that promote invasion (‘novel weapons’)^9^ and modification of local above- and below-ground environments^7^,^9^. One particularly successful plant invader, *Echium plantagineum* L., is self-incompatible in its native range but purportedly became self-compatible after introduction to Australia^10^, providing further evidence for the hypothesis of increased colonizing ability. Although uniparental reproduction may result in inbreeding depression^11^, annual self-compatible invaders may operate more independently from conspecifics and ancestral pollinators^10^.

Evaluation of evolutionary genetics of invaders is critical to develop a better understanding of the mechanism(s) associated with invasion success. With regards to successful plant invasion, sufficient levels of genetic diversity are typically required for species persistence and evolution in a dynamic environment^12^,^13^. High levels of genetic diversity may contribute to adaptive potential and resistance to environmental stress, including management practices. However, a plant invader commonly establishes initially with limited genetic variation, as most invasions are derived from small founder populations^14^. Invader populations may subsequently increase in genetic diversity over time via introduction of new genotypes, rapid evolution and/or cross-species hybridization^7^,^15^. Dlugosch and Parker^16^ highlighted the importance of multiple introductions and adaptive evolution for species invasion. For example, the house sparrow and European starling became problematic only after multiple introductions into North America^13^.

Numerous studies support a negative relationship between plant monoploid genome size and invasiveness^17^,^18^,^19^,^20^,^21^. According to “the large genome constraint hypothesis”, smaller genomes are associated with shorter life cycles, smaller seed, greater specific leaf area and higher photosynthetic rates^17^,^18^. Cytogenetic analysis of 156 weedy and 2685 non-weedy species indicated that weedy species tend to have smaller genome size (3.79 pg) compared to non-weedy species (12.14 pg)^19^. Very small genomes (1 C < 1.40 pg) are very common in the most invasive plant species^21^.

A direct comparison of the genetics and invasion ecology of both successful and less successful plant invaders introduced at similar timeframes to the same or similar location(s) could result in significantly enhanced understanding of the mechanisms that drive invasion success. Therefore, the congenerics *E. vulgare* L., commonly called Viper’s bugloss, and *E. plantagineum*, known regionally as Paterson’s curse or Salvation Jane, were chosen as model species in this study because of their similar introduction history, morphology, reproduction and dispersal^22^. Both species originated in the Mediterranean and have since naturalized in Africa, America, Asia, Europe and Oceania^23^,^24^. *Echium vulgare* is now commonly encountered in Europe and Canada^24^,^25^ but in Australia is restricted to the south-eastern states of South Australia (SA), New South Wales (NSW), Victoria (VIC) and Tasmania (TAS)^25^. In contrast, *E. plantagineum* is an economically important weed in Australia^26^ and has invaded 33 million hectares across southern and western Australia, with an estimated annual economic impact of $250 million^27^. Both *Echium* species are drought tolerant, can produce up to 10,000 seeds per plant and rely on mammalian activity for dispersal^23^,^24^. Unfortunately in Australia, ‘Paterson’s curse’ has sometimes been used as a common name for either *E. vulgare* or *E. plantagineum*^28^ so the extent of distribution following establishment in the 1800 s is potentially unclear^29^.

Depending on seasonal growing conditions, *Echium plantagineum* can exist either as an annual or biennial. It was reportedly introduced to Australia in the mid-1800 s as an ornamental plant^23^, but quite possibly was repeatedly introduced with the direct importation of merino sheep from northern Spain^30^. *Echium plantagineum* is a native of the Iberian Peninsula and today can be found sporadically throughout the Mediterranean region. In contrast, *E. vulgare* is reported to be a biennial or short-lived perennial, and is widespread across temperate regions of Europe. It is thought to have been introduced to Australia around 1820^29^.

*Echium vulgare* and *E. plantagineum* produce two interesting groups of secondary metabolites important in plant defence: pyrrolizidine alkaloids synthesized in above-ground plant tissues and organs, and naphthoquinones produced in living roots and root hairs^31^,^32^,^33^,^34^. Pyrrolizidine alkaloids play critical roles in plant defence against grazing herbivores and are present in high concentrations in both *E. vulgare* and *E. plantagineum*, thus contributing to livestock toxicity across southern Australia due to their direct consumption^23^,^32^. The roots of *E. plantagineum* and *E. vulgare* also produce high concentrations of naphthoquinones^34^, red-coloured compounds referred to as shikonins that are also produced by roots of other members of the Boraginaceae^35^. Shikonins exhibit potent antimicrobial, antifungal, and phytotoxic properties and are frequently used as biomedicinals in Eastern medicine^35^. In Australia, exposure to stressful conditions is associated with enhanced production of shikonins and pyrrolizidine alkaloids in *E. plantagineum*, with increased concentrations observed in plants collected from warmer, drier locations^32^,^33^. Other Boraginaceae including *Lithosperum* L. and *Arnebia* Forssk. also produce shikonins^35^. Our recent studies suggest that both families of metabolites contribute to plant defence and may serve as important ‘novel weapons’ in the invasion process^36^.

Past studies of *E. plantagineum* and *E. vulgare* in Australia have focused mainly on pollination ecology and floral nectar production related to quality of commercially produced honey^37^. However, specific information on comparative morphology^38^, phenology^39^,^40^,^41^,^42^, genetics, and biology^23^,^24^ is limited. Both species have been sparingly included in broader phylogenetic studies of *Echium* spp.; thus limited information is available regarding their contemporary spatial distributions^11^,^43^,^44^,^45^. The most recent study of genetic diversity of *E. plantagineum* in Australia used isozyme markers to study diversity and suggested a similar level of genetic diversity between Australian and native Iberian populations^46^. As polymorphisms detected by isozyme markers vary among tissues, growth stages and environments^47^, and methods of specimen preservation often impact isozyme analyses, further studies are warranted.

To shed additional light on the mechanisms of invasion success of these two congeneric species in Australia, a series of field surveys was performed across southern Australia in locations where both species are now naturalized^27^. Specimen records from Australian herbaria were evaluated to gain an understanding of the historical introduction of each species to Australia. Geographically distinct populations of both species were surveyed for local climatic conditions and coexisting plant diversity. The hypothesis of evolution of increased competitive ability was tested by measuring qualitative and quantitative differences in secondary metabolite production. We also hypothesized that the invasive *E. plantagineum* has smaller monoploid genome size and higher level of genetic diversity compared to naturalized *E. vulgare*.

## Results

### Geographic distribution in Australia

**Figure 1:**
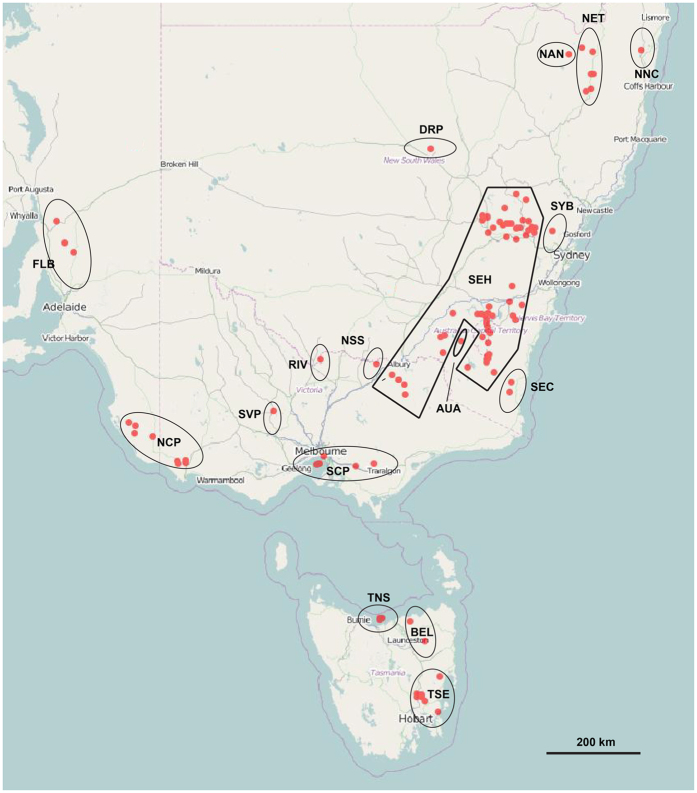
Distribution of *Echium vulgare* in Australia. Red dots indicate the location of herbarium specimen records of *E. vulgare*. Dots enclosed by solid lines indicate records obtained from the same biogeographic region. See Table 1 for biogeographic regional codes. Image provided by Australia’s Virtual Herbarium^48^.

Results obtained from three seasons of field surveys conducted in southern Australia are in general agreement with historical herbarium records obtained for both *Echium* species in Australia’s Virtual Herbarium (AVH)^48^. *Echium plantagineum* was found to be widely distributed across southern Australia (Supplementary Fig. S1a). However, *E. vulgare* was found only sporadically, and was narrowly restricted to the South Eastern Highlands (SEH) biogeographic region (Supplementary Figs S1b and S2). We noted 1376 and 174 AVH records of *E. plantagineum* and *E. vulgare*, respectively, in Australia. *Echium plantagineum* is widely distributed from eastern Queensland (QLD) to Western Australia (WA), being recorded in Brigalow Belt North, QLD and also across nearly all of the biogeographic regions (around 40) in NSW, VIC, TAS and SA to Carnarvon, WA. In contrast, *E. vulgare* was restricted to 17 biogeographic regions, with most records coming from one biogeographic region, SEH, which accounts for 59.2% of total records in Australia (Fig. 1). This species was reported sporadically in only four biogeographic regions since 2000: New England Tablelands (NET), SEH, Ben Lomond (BEL), and Tasmanian South East (TSE) (Table 1, Fig. 1 and Supplementary Fig. S2). Historical records of *E. vulgare* also indicate past occurrences in TAS, southeastern NSW and VIC, where summer rainfall is more common, elevation typically exceeds 400 m and recorded winter temperatures are below 3 °C. In contrast, some records were noted from SA, western NSW and VIC, where summer rainfall is limited, elevation is lower than 300 m and winter temperatures are generally warmer (Table 1).

### Impact of *Echium* invasion on plant biodiversity in Australia

From 2011 onwards, it proved particularly difficult to find established sites of infestation for *E. vulgare* across southern Australia. Four sites infested with *E. vulgare* were noted and analysed; the density of *E. vulgare* ranged from 2–67 plants m^−2^, and averaged 27.0 ± 14.3 plants m^−2^ (mean ± SEM). In contrast, sites infested with *E. plantagineum* were easily detected and numerous; the density of *E. plantagineum* in the most heavily infested quadrat was 275 plants m^−2^, and averaged 80.9 ± 19.3 plants m^−2^ for the 17 sampled locations. Plant biodiversity decreased when *E. plantagineum* was present in quadrats but not when *E. vulgare* was present. The number of all other species per quadrat decreased from 6.6 ± 0.7 to 4.6 ± 0.5 when *E. plantagineum* was present (*P* < 0.01), whereas the corresponding values for *E. vulgare* were 5.3 ± 1.0 vs 4.0 ± 0.7 (difference not significant at *P* = 0.05). The density of other plants was more heavily impacted by the presence of *E. plantagineum*. These values declined from 1271.2 ± 219.8 m^−2^ in quadrats where *E. plantagineum* was absent to 689.6 ± 130.2 m^−2^ when *E. plantagineum* was present (*P* < 0.01); corresponding values for *E. vulgare* were 1018.8 ± 240.7 m^−2^ and 1043.8 ± 82.5 m^−2^, respectively (*P* > 0.5). These measures of biodiversity are not directly comparable because of the higher densities of *E. plantagineum* observed, but when restricting the analysis to quadrats where *E. plantagineum* spanned a similar range of densities to *E. vulgare* (13–76 plants m^−2^, *n* = 11), a significant decrease in number of other plants was still observed with increased density of *E. plantagineum* (1112.3 ± 260 uninfested with *E. plantagineum* vs 552.1 ± 176.9 infested, *P* < 0.05).

### Pyrrolizidine alkaloid content

**Figure 2:**
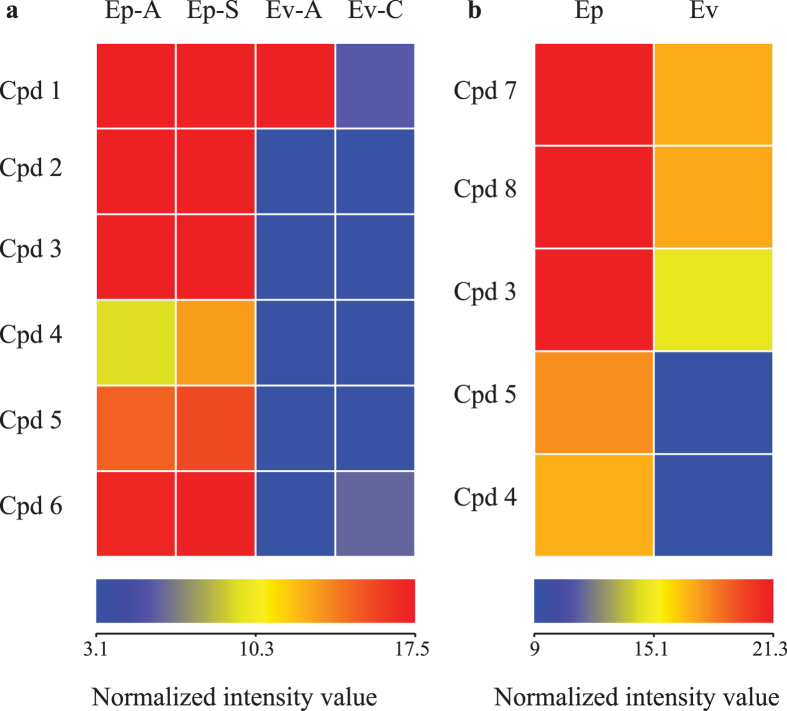
The relative abundance of pyrrolizidine alkaloids and their *N*-oxides extracted from *E. plantagineum* (Ep) and *E. vulgare* (Ev) foliar tissue, averaged over three biological replications for each treatment. Data was normalized by log_2_ transformation. Both species were grown (**a**) under uniform glasshouse condition or (**b**) at the same field sites near Bathurst. Pyrrolizidine alkaloids were significantly more abundant in Ep as tested by one-way ANOVA (*P* < 0.05). Ep: *Echium plantagineum*, Ev: *E. vulgare*; Ep-A: *E. plantagineum* collected from Adelong; Ep-S: *E. plantagineum* collected from Silverton; Ev-A: *E. vulgare* collected from Adaminaby; Ev-C: *E. vulgare* collected from Cooma. Please refer to Table S1 for the name of the compounds.

Metabolic profiling (using ultra high pressure liquid column chromatography coupled to time of flight mass spectrometry, or UPLC MS QToF) of foliage from geographically diverse field- and glasshouse-grown plant populations of both species resulted in detection of 17 pyrrolizidine alkaloids in *E. plantagineum* leaf extracts and up to 16 pyrrolizidine alkaloids in *E. vulgare* shoot extracts (Table S1). This corresponds with recent studies noting up to 17 pyrrolizidine alkaloids in *Echium spp*. shoot extracts^32^. Of note is the finding that pyrrolizidine alkaloids occurred in *E. plantagineum* at levels up to three times those observed in *E. vulgare*, a result confirmed both in controlled glasshouse conditions and in field sampling when species ranges overlapped near Bathurst NSW (Fig. 2). Three pyrrolizidine alkaloids were consistently less abundant in *E. vulgare* in all environments: 7-O-acetyllycopsamine-*N*-oxide B, 3′-O-acetylechiumine-*N*-oxide and 7-O-acetyllycopsamine.

### Genome size and genetic diversity

**Figure 3:**
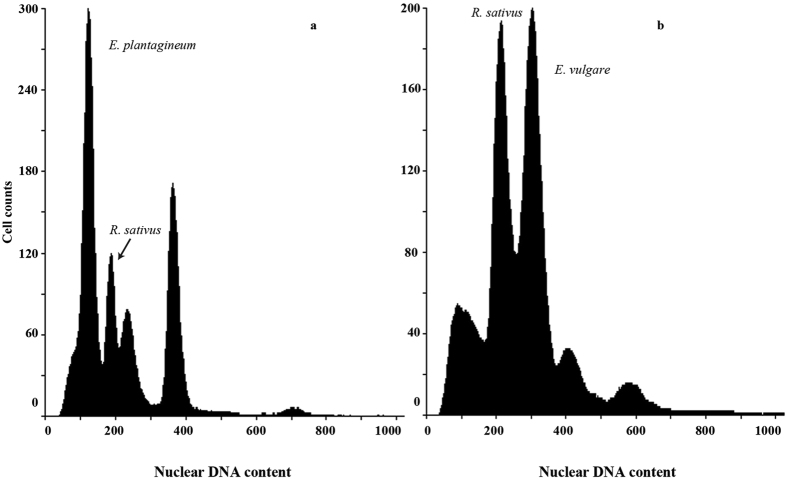
Flow cytometry histograms of *E. plantagineum* (**a**) and *E. vulgare* (**b**) using radish (*Raphanus sativus* 1 C = 0.55 pg) as an internal reference.

Monoploid genome size (presented as 1 C value) of *E. vulgare* ranged from 0.41 to 0.45 pg (mean: 0.43 ± 0.003 pg), while the 1 C value of *E. plantagineum* ranged from 0.30 to 0.39 pg (mean: 0.34 ± 0.002 pg) (Table 2 and Fig. 3). Results obtained are consistent with the previously reported ploidy level of both species in Europe (2n = 32 for *E. vulgare* and 2n = 16 for *E. plantagineum*)^49^. Neither species showed a change in DNA content with variation in ploidy, nor was there any apparent difference in genome size in geographically distinct locations/populations for each species.

PCR and sequencing analysis were 100% successful for all samples at targeted gene regions; 154 sequences were generated for each gene region under scrutiny. Alignments were truncated to 636, 280, 469 and 399 bp for ITS, *trn*H-*psb*A spacer, *trn*L intron and *trn*L-*trn*F spacer, respectively. Four alleles were detected in the nuclear ITS region and two haplotypes were found in the concatenated chloroplast regions of *E. vulgare*; the corresponding values detected for *E. plantagineum* included two alleles and 12 haplotypes (Table 3).

*Echium vulgare* showed a similar level of nucleotide (*π* = 0.0015)^50^ and haplotype (*h* = 0.5444) genetic diversity^50^ in the nuclear region (ITS) to that of *E. plantagineum (π* = 0.0008, *h* = 0.4990) (Table 3). However, considerably lower genetic diversity was detected in the chloroplast regions of *E. vulgare (π* = 0.0014, *h* = 0.3800) compared to *E. plantagineum (π* = 0.0021, *h* = 0.7661).

**Figure 4:**
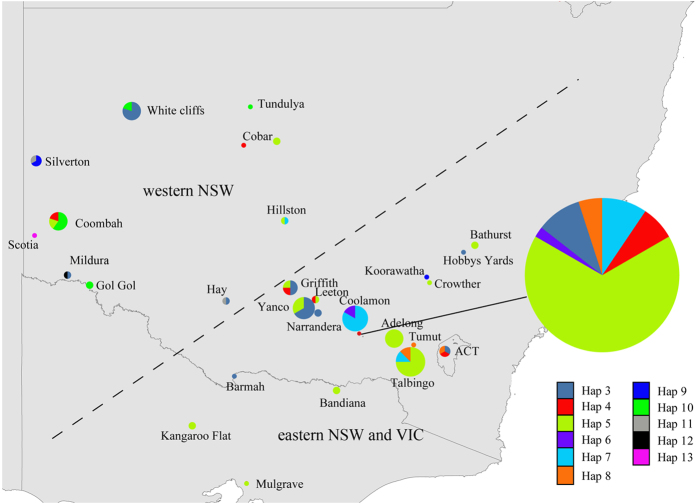
There are two spelling problem in this image. Please use the revised image uploaded with this proof. The dashed line separates southern Australia into eastern NSW and VIC, and western NSW. (specific haplotypes Hap 6 and 8 are found in eastern NSW and VIC, and Hap 10, 11, 12 and 13 in western NSW, respectively. This map is a derivative of “State and Territory ASGC Ed 2011 Digital Boundaries in ESRI Shapefile Format” sourced from the Australian Bureau of Statistics, used under CC BY 2.5 (https://creativecommons.org/licenses/by/2.5/au/) and modified using ArcGIS 10.3.1 software by Esri (http://www.esri.com) and Adobe Illustrator CS5.

Evidence of regional chloroplast population structure in *E. plantagineum* was noted. The distribution of *E. plantagineum* chloroplast haplotypes (*n* = 12) showed strong indication of geographic sorting between western NSW and southeastern Australia (Fig. 4), as indicated by shifts in frequency of haplotype 5 (Supplementary Fig. S3), observed as prevalent in eastern NSW and VIC (54.4%), but less so in western NSW (15.4%). Haplotypes 10–13 were not observed in eastern NSW and VIC, but represented 42.3% of the haplotypes found in western NSW. In addition, haplotypes 6 and 8, present at low frequencies in eastern NSW and VIC (2.2 and 5.6%, respectively), were not found in western NSW. A population pairwise *F*_st_ test^51^ showed a significant (*F*_st_ = 0.13, *P* < 0.001) difference between western NSW and eastern NSW and VIC, which strongly suggests the presence of genetic structure. This population structure was not supported at the nuclear ITS gene, where structure was evaluated using an *F*_st_ test (*F*_st_ = −0.02, *P* = 0.85) and 95% parsimony network analysis further indicating that the two nuclear ITS alleles in *E. plantagineum* were generally present at similar frequencies across sampled regions (Supplementary Fig. S4a). The *E. plantagineum* chloroplast network analysis suggested no apparent phylogenetic basis for haplotype sorting among regions (Supplementary Fig. S4b). Interestingly, one rare haplotype, 14, was unique to WA.

## Discussion

*Echium plantagineum* was first recorded in Australia in MacArthur Garden, located in Camden, NSW (near Sydney, NSW) and introduction from England as an ornamental is postulated^23^. It is uncertain, however, whether this introduction event resulted in later escape and naturalisation. In Australia, at least three naturalisation events of *E. plantagineum* have been documented, one near Albury (NSW), one in Gladstone (near Port Pirie, SA) and one in WA, all in the 1880 s^52^. Considering the similar timing of these events and the great distance between these Australian locations, it is likely that multiple introductions of *E. plantagineum* occurred^29^,^52^. Distribution of the 12 observed chloroplast haplotypes in Australia noted from our analyses is well-aligned with these reported naturalisation events. Regional specific haplotypes were detected in eastern NSW and VIC (haplotypes 6 and 8), western NSW (haplotypes 10–13) and WA (haplotypes 14) (Figs 4, S3 and S4). Although 90 individuals were sampled, samples from eastern NSW and VIC represented only 7 of the 12 detected haplotypes of Australian *E. plantagineum*, with two specific haplotypes (haplotypes 6 and 8) occurring near Albury, NSW. In contrast, the western part of NSW, located between the SA and NSW introduction events, contained nearly all of the *E. plantagineum* haplotypes (9 out of 12, except haplotypes 6, 8 and 14) detected in this survey. It is possible that additional sampling in SA might result in the recovery of additional or specific haplotypes (such as 10–13). The *F*_st_ test revealed a significant population structure in chloroplast DNA (*P* < 0.001) but not in nuclear DNA (*P* = 0.85). Lack of population structure at ITS may be caused by the paucity of available polymorphism at ITS of Australian *E. plantagineum* and/or higher migration rates of nuclear DNA in contrast to chloroplast DNA. Plastid DNA is maternally inherited in angiosperms^53^, which means the cpDNA of *E. plantagineum* and *E. vulgare* can move only by seed distribution, while the gene flow of the nuclear region can be attributed to both seed and pollen dispersal^54^.

*Echium plantagineum* is apparently less prone to genetic bottlenecks because of its greater adaptability across a variety of habitats. Multiple introductions of *E. plantagineum* to Australia, evidenced by the population structure in south-eastern Australia, may also have contributed to its high genetic diversity. High genetic diversity is associated with invasion success for many plant species^7^,^55^,^56^,^57^,^58^. Careful management of each species in local regions may be critical in future years to avoid seed dispersal across Australia and limit out-crossing that may result in further enhancement of genetic diversity among distinct regional genotypes within each species. In addition, considering that *E. vulgare* is a weed of importance in Europe^25^ and Canada^24^, it will also be critical to avoid new introductions of *E. vulgare* into Australia that might increase the number of genotypes post-introduction.

The invasive species *E. plantagineum* possesses a distinctly smaller genome size than the non-invasive *E. vulgare* (Table 2), which supports the large genome constraint hypothesis^17^. A small monoploid genome size (1 C < 1.40 pg) is often found at high frequency in invasive species^21^ and is normally also associated with reduced generation time and seed mass and increased relative growth rate and seed numbers^21^,^59^. However, studies on *Phragmites australis* (Cav.) Trin. ex Steud. suggested that smaller genome size can also potentially reduce plant fitness and defence^60^. There was no significant difference in genome size among 93 invasive and naturalized species in the Czech Republic^61^, which suggested that small monoploid genomes may be critical for the initial settlement of alien species but less important after establishment^21^. Small genome is also correlated with an annual life cycle in some plant genera, including *Veronica* L.^62^ and *Sorghum* Moench^63^. It is not clear in Australia whether genome size is related to the persistent spread of weedy features and/or phenology/life cycle in the genus *Echium*. When compared with previously reported data on 13 other *Echium* species^20^, monoploid genome size of annual *Echium* species (1 C DNA content range: 0.30–0.32 pg) is considerably smaller than the perennial *Echium* species (1 C DNA content range: 0.41–0.43 pg). However, as data from only two annual *Echium* species (*E. bonnetii* Coincy and *E. plantagineum*) has been published, it is speculative to generalise that reduced genome size is associated with a shorter life cycle in the genus as a whole. Polyploidy, often reported as occurring in invasive weeds and suspected of enabling certain species to gain plasticity associated with specific habitat and resource requirements resulting in adaptation to broader environmental parameters^64^, has apparently not been a factor contributing to variable success of *E. plantagineum* and *E. vulgare* in colonising Australia (Table 2).

*Echium plantagineum* in Australia exhibits a considerably shorter life cycle and produces greater leaf area than does *E. vulgare*^42^, and also produces larger seeds (3.6–3.9 mg per seed compared to 2.5 mg per seed for *E. vulgare*)^23^,^24^. A shorter life cycle may facilitate the broader adaptation of *E. plantagineum* to diverse and variable climatic conditions and thereby facilitate escape from environmental stress. Both species are capable of producing similar numbers of seeds per plant, but a shorter life cycle has potentially enabled *E. plantagineum* to produce more seed over time, as both species are monocarpic^15^. In addition, *E. plantagineum* tended to suppress the number of other species growing in close proximity, as suggested by the density of other plants in quadrats where these species were sampled compared with nearby quadrats where *Echium* spp. were absent. *Echium plantagineum* also appeared to achieve greater overall densities than *E. vulgare*, but this result is not definitive because of the small number of observed sites infested with *E. vulgare*.

Recent records of *E. vulgare* were found in only four biogeographic regions (NET, SEH, BEL and TSE), where cold winters and reliable summer rainfall (or high humidity) were common. *Echium vulgare* has also been reported in six biogeographic regions of SA, western NSW and VIC with warmer winter temperatures and limited summer rainfall (Table 1). A comparison of the recent decade (2005–2014) with the previous 50 years (1955–2004) of climate data (Table S2) shows a clear trend toward increased winter temperatures and more frequent summer rainfall events. Increased summer rainfall is likely to promote germination of both species from the existing seed bank, but probably more so for *E. plantagineum* since its existing seedbanks are likely more plentiful as discussed aboved^24^. In addition, without exposure to cooler winter temperatures for vernalisation, *E. vulgare* may become increasingly less abundant in Australia.

A high rate of germination (>40%) is typically achieved at warmer soil temperatures ranging between 20–30 °C in late spring and summer for *E. vulgare*, or between 10–30 °C in early spring and summer for *E. plantagineum*^42^. Germination of *E. plantagineum* normally occurs after spring and summer rainfall events in Australia^23^ whereas optimal germination conditions for *E. vulgare* in the field are associated with higher soil temperature and moisture availability to support maximal emergence; the seedlings of this species therefore emerge weeks to months later than those of *E. plantagineum* in the same biogeographic region^24^,^42^. *Echium plantagineum* is also highly resistant to water deficit. Most (57%) *E. plantagineum* seedlings survived after 2–4 weeks under severe moisture stress in Albury (southeastern NSW)^23^, and we have also observed extreme tolerance of this species to moisture deficit after withholding water for up to 3 weeks in controlled environment experimentation (unpublished data). We do not know of comparable tests for *E. vulgare*, but in experimentation performed in Canada, only 18% of seedlings survived their first year of establishment and only 5% of all established seedlings reached reproductive maturity, with many seedlings experiencing mortality due to drought following emergence^24^,^42^. In inland Australia, rainfall typically occurs more frequently in winter months, when soil temperatures are generally not high enough to support the emergence of *E. vulgare*. Both summer and early autumn rainfall events in southern and western Australia may induce germination, but are normally followed by severe periods of drought, which could potentially result in high mortality of *E. vulgare* seedlings. *Echium vulgare* also has a vernalisation requirement and requires low temperatures “throughout the winter” to induce flowering in potted plants, while warm summers were necessary for vegetative growth^42^. Without intermittent exposure to cooler winter conditions, *E. vulgare* has been observed to remain as a vegetative rosette for 10 years in a continuously warm environment^24^. These factors would undoubtedly result in lower reproductive success of *E. vulgare* in much of inland Australia.

The higher abundance of pyrrolizidine alkaloids in the foliage of *E. plantagineum* may limit feeding by animals, both vertebrate and invertebrate, on this species *vis-à-vis E. vulgare*. Specialist insects are able to successfully feed on *Echium* spp. and other plant species containing pyrrolizidine alkaloids, but most generalist insects lack the ability to sequester or detoxify these compounds^65^. The presence of pyrrolizidine alkaloids is readily detected by native or unadapted insect herbivores^66^, causing these insects to look elsewhere for feed after sampling foliage. Livestock are known to feed on *Echium* spp. when other species are scarce, but grazers are also able to detect the presence of pyrrolizidine alkaloids and would thus typically avoid feeding on plants containing them. The greater abundance of alkaloids in *E. plantagineum* is likely to have a stronger protective effect than the reduced levels found in *E. vulgare*. In addition to foliar alkaloids, naphthoquinones (shikonins) present in the roots of *E. plantagineum* and *E. vulgare* are active against a range of biotic threats including microbiota and neighbouring plants, and their variable production may also contribute to the differential invasion success of these two species^31^,^32^,^33^. Although glasshouse grown *E. vulgare* plants show higher abundance of shikonins than does *E. plantagineum*, drought conditions experienced in the field may stimulate increased production of shikonins by *E. plantagineum* to a greater extent than *E. vulgare*^31^,^32^,^33^, suggesting that the former may be better defended against herbivores and more competitive under stressful conditions.

Herbarium records were essential in this study for documentation of the historical dynamics of dispersal of the weedy invaders *E. plantagineum* and *E. vulgare* across Australia^42^. However, misidentification of *Echium* species was and continues to be very common in Australia^29^, and field surveys are clearly required to verify the current infestation rate of each species. Two multi-year surveys performed over 2011–2015 confirmed the previous records of *E. plantagineum* invasion across southeastern Australia. However, in contrast to past reports, *E. vulgare* was found only sporadically in the SEH biogeographic region in eastern NSW during this period. *Echium vulgare* was generally observed near the edges of roadsides in the southern highlands at higher elevations, but at very low densities. In contrast, *E. plantagineum* was found broadly distributed along roadsides, railroad tracks, in stockyards and grazing lands, but was normally at very high densities, including monocultural stands, and in larger populations.

In summary, greater success of *E. plantagineum* in contrast to that of *E. vulgare* in colonising the Australian continent since introduction in the 1800 s corresponds with variation in a number of attributes between the two species: *E. plantagineum* has 1) a better match between its phenology and the Mediterranean type climate encountered across much of Australia, 2) greater drought tolerance, 3) greater genetic diversity and smaller genome size, and 4) greater abundance of defensive and potentially offensive secondary compounds. The invasion history of this genus in Australia thus provides support for several (non-mutually exclusive) hypotheses previously proposed to explain the ability of plant species to invade new territories.

## Materials and Methods

### Current and historical survey of *E. vulgare* and *E. plantagineum* distribution

The distribution of *E. plantagineum* and *E. vulgare* was initially reviewed by examination of herbarium records available from the AVH^48^. The identity of specimens falling outside of the expected distribution of either taxon was re-examined, and identifications corrected where necessary. A large field survey for presence of *E. plantagineum* and *E. vulgare* was conducted in the spring of 2011, 2012 and 2013 across southeastern Australia covering 76 locations aligned with three longitudinal transects^32^. Additional survey points were included in the Riverina region (Fig. S1) to survey additional geographically distinct populations of each species. As per the Interim Biogeographic Regionalisation for Australia (IBRA) survey, Australia is currently divided into 89 biogeographic regions (Fig. S2) according to climate, geology, landform, species and native vegetation (http://www.environment.gov.au/land/nrs/science/ibra). For regional climatic analyses, average temperature and annual rainfall for each biogeographic region of collection were obtained from the Spatial Data Analysis Network at Charles Sturt University, Wagga Wagga, NSW.

### Ecology survey - impact of infestation on *Echium* spp. growth and local plant biodiversity

An ecological field survey was conducted at 17 and four sites of *E. plantagineum* and *E. vulgare*, respectively (Table S3), in the summer of 2013 and 2014 to investigate the impact of the establishment of these two invaders on local plant biodiversity. For both species, data were collected from two 1 m × 1 m quadrats at each location. The number of *Echium* sp. individuals, number of other plant species, and total number of other plants present in quadrats were recorded. Means were compared with the Wilcoxon signed-rank test (Statistix ver. 9.0^67^) because ecological data did not meet the requirements for ANOVA.

### Comparison of genome size

*Echium vulgare* leaf tissues were collected from the four known geographically distinct locations of *E. vulgare* infestation in the SEH biogeographic region, while *E. plantagineum* seedlings from 11 locations were obtained after seed germination (Table 2). Fresh leaf tissue from numerous individuals (9–15 per population depending on successful germination and establishment) were collected and analysed within 48 hours, depending on availability. A total of 60 *E. vulgare* and 140 *E. plantagineum* samples from geographically distinct locations were analysed. Samples were prepared for flow cytometry analysis according to Loureiro, *et al*.^68^ using WPB nuclei isolation buffer. *Raphanus sativus* L. (red globe radish) was used as internal reference for assessment of genome size in *E. vulgare* and *E. plantagineum*^20^. Three samples from each location were examined individually by comparison to the radish genome using a Gallios Flow Cytometer (Beckman Coulter, USA), and any additional samples from each site were pooled for analysis. Three independent repetitions were performed for each sample on separate days, with at least 5,000 nuclei being analysed each time. Genome size of samples from each location was compared using an unbalanced ANOVA (location as factor) with GenStat 17^th^ edition^69^.

### Comparison of genetic diversity

A total of 25 *E. vulgare* and 129 *E. plantagineum* plan samples were used for genetic diversity analysis (Table S4), which included preserved herbarium voucher specimens provided by Brendan J. Lepschi of the Australian National Herbarium (four *E. vulgare* and 20 *E. plantagineum*). Samples of *E. plantagineum* and *E. vulgare* originated from widely distributed locations across the known endemic range of each species in southeastern Australia (Fig. S1). In addition, Wagga Wagga (NSW) experienced several large outbreaks of *E. plantagineum* which were also monitored and included in sampling. This region was therefore considered a hotspot of diversity following preliminary evaluation and a total of 42 samples were collected from Wagga Wagga NSW for additional haplotype analysis. Several samples from WA, TAS, and the Northern Territory were also sequenced.

Genomic DNA isolation, PCR, sequencing and alignment procedures were performed as described previously^70^. Samples were sequenced for one nuclear gene (ITS) and three chloroplast DNA regions (*trn*H-*psb*A spacer, *trn*L intron and *trn*L-*trn*F spacer). A 25 bp portion was discarded from the *trn*L intron sequence alignment due to low sequencing quality caused by a homopolymeric region (polyA and polyT) present in the sequence. Indels were coded as single mutations as described previously^71^ (Appendix 1). In addition, a 4 bp inversion region in *trn*H-*psb*A spacer^72^ was also coded as a single mutation^73^. The alignments of three linked chloroplast DNA regions were concatenated using FABOX^74^. Heterozygotes of nuclear ITS sequences were phased into two separate sequences via PHASE 2.1^75^, using 1000 iterations, 10 thinning intervals and 1000 burn-in iterations. The algorithm was run five times using the “-x option” to obtain accurate estimates^75^. All sequences reported in this study have been deposited in the GenBank database under the GenBank accession numbers KX012007-KX012622.

Nucleotide (*h*) and haplotype (*π*) genetic diversity estimates^50^ were calculated within species using ARLEQUIN ver. 3.5^76^, and 95% statistical parsimony network analyses was performed to investigate the nuclear and chloroplast DNA genealogical relationships in *E. plantagineum* using TCS ver. 1.21^77^. Network analysis was not performed for *E. vulgare* due to the limited number of haplotypes detected in our sampling.

### Metabolic profiling, UPLC MS QToF and data analysis of leaf extracts in *E. plantagineum* and *E. vulgare*

Both species were evaluated under uniform glasshouse conditions and near identical field conditions in neighbouring collection sites to assess the production of pyrrolizidine alkaloids. Seeds of *E. plantagineum* were collected from Adelong (N: −35.296, E: 148.057) and Silverton (N: −31.883, E: 141.216), NSW, while seeds of *E. vulgare* were collected from Adaminaby (N: −35.995, E: 148.791) and Cooma (N: −36.140, E: 149.200), NSW. Plants were cultivated in a glasshouse as described previously^32^ using a randomised block design experiment (three blocks) and harvested sequentially by blocks when *E. plantagineum* plants were flowering and *E. vulgare* were at the rosette stage. *Echium vulgare* did not flower likely due to its perennial growth habit and lack of vernalisation^32^. Field plants of both species were collected from a roadside population near Bathurst, NSW (N: −33.463, E: 149.476) at flowering stage. To our knowledge, this is the first reported site where both species were co-located at the same site. Leaves were combined from three or four plants in the field and glasshouse experiment, respectively, to obtain a composite sample of 4.0 g of foliar tissue for extraction. Foliar tissue extraction, solid phase extraction, UPLC MS QToF analysis and statistical analysis were performed as previously described^32^.

## Additional Information

**How to cite this article:** Zhu, X. *et al*. Ecology and genetics affect relative invasion success of two *Echium* species in Southern Australia. *Sci. Rep.* **7**, 42792; doi: 10.1038/srep42792 (2017).

**Publisher's note:** Springer Nature remains neutral with regard to jurisdictional claims in published maps and institutional affiliations.

## Supplementary Material

Supplementary Info File

Supplementary Information

## Figures and Tables

**Table 1:** Climatic conditions experienced (1955–2014) in Australian biogeographic regions supporting *Echium vulgare* (Supplementary Fig. S2).

Biogeographic region^1^	Code	Number of records^2^	Elevation (m)^3^	Latest record^2^	Mean minimum temperature of the coolest month (°C)^4^	Mean summer rainfall (mm)^4^	Mean winter rainfall (mm)4
TAS and Southeastern NSW and VIC							
South East Highlands	SEH	103	400–1396	2015	0.75	68.55	84.22
Tasmanian South East	TSE	16	8–20	2009	2.39	65.58	141.99
New England Tablelands	NET	6	900–1300	2008	0.79	107.84	48.68
Tasmanian Northern Slopes	TNS	5	280	1984	2.29	55.66	77.35
Ben Lomond	BEL	2	10–280	2011	1.81	64.01	119.03
South East Corner	SEC	3	50	1976	2.40	74.33	70.67
NSW South Western Slopes	NSS	1	150	1981	2.46	51.33	56.69
Australian Alps	AUA	1	1480	1988	−1.62	85.34	139.41
Nandewar	NAN	1	580	1954	1.87	88.25	43.79
NSW North Coast	NNC	1	90	1976	4.00	142.07	62.17
Sydney Basin	SYB	1	260	1998	3.83	99.91	60.04
SA and western NSW and VIC							
South East Coastal plain	SCP	11	90–230	1976	5.13	49.09	74.15
Naracoorte Coastal Plain	NCP	10	5–60	1979	5.74	24.93	82.26
Riverina	RIV	6	90	1973	3.54	31.73	36.48
Flinders Lofty Block	FLB	5	278	1990	3.94	24.16	31.64
Darling Riverine Plains	DRP	1	200	1976	4.36	56.12	29.18
Southern Volcanic Plain	SVP	1	300	1918	4.42	39.52	66.80

^1^Biogeographic regions were defined by Interim Biogeographic Regionalisation for Australia (please see Fig. S2); ^2^*E. vulgare* records were obtained from Australia’s Virtual Herbarium (avh.chah.org.au). ^3^Elevation data were obtained from AVH or estimated from the elevation of the nearest city or town. ^4^Climate data were provided by the Spatial Data Analysis Network of Charles Sturt University (SPAN; https://www.csu.edu.au/research/span/home).

**Table 2:** Genome size of Australian *E. vulgare (Ev*) and *E. plantagineum (Ep*) as estimated by flow cytometry using genome size of radish (*Raphanus sativus* 1 C = 0.55 pg) for standard comparison^68^.

Species	Ploidy level	Location of collection^1^	Genome size: 1 C (pg)^2^	Peak CV (%)^3^	Number of samples evaluated
*Ev*	2n = 32	Adaminaby	0.43 ± 0.003	5.35	15
*Ev*	2n = 32	Cooma	0.43 ± 0.007	6.04	15
*Ev*	2n = 32	Mt. Denison	0.44 ± 0.005	6.13	15
*Ev*	2n = 32	Numeralla	0.43 ± 0.004	5.56	15
*Ep*	2n = 16	Bandiana	0.34 ± 0.005	10.97	15
*Ep*	2n = 16	Coombah	0.35 ± 0.004	11.05	13
*Ep*	2n = 16	Gol Gol	0.34 ± 0.004	11.51	13
*Ep*	2n = 16	Kangaroo Flat	0.33 ± 0.001	11.92	10
*Ep*	2n = 16	Narrandera 1	0.33 ± 0.005	11.18	15
*Ep*	2n = 16	Talbingo	0.36 ± 0.012	11.53	9
*Ep*	2n = 16	Wagga Wagga 1	0.32 ± 0.006	11.90	15
*Ep*	2n = 16	Wagga Wagga 2	0.33 ± 0.002	10.88	15
*Ep*	2n = 16	Wagga Wagga 3	0.34 ± 0.005	11.62	15
*Ep*	2n = 16	Wagga Wagga 4	0.33 ± 0.002	11.06	11
*Ep*	2n = 16	White Cliffs	0.35 ± 0.005	11.16	9

^1^Please refer to Table S4 for GPS coordinates of each location; ^2^Values are given as mean and standard error of mean; ^3^coefficient of variation of sample.

**Table 3:** Genetic diversity of Australian *E. vulgare* and *E. plantagineum*, as estimated by allele and haplotype (hap) numbers, nucleotide (*π*) and haplotype (*h*) diversity.

DNA regions		*Echium vulgare*	*Echium plantagineum*
ITS	allele	4	2
	*π*	0.0015 ± 0.0011	0.0008 +/− 0.0007
	*h*	0.5444 ± 0.0649	0.4990 +/− 0.0072
Chloroplast	hap	2	12
	*π*	0.0024 +/− 0.0015	0.0029 +/− 0.0017
	*h*	0.3800 +/− 0.0913	0.7661 +/− 0.0298
