# Erratum: Ecology and genetics affect relative invasion success of two *Echium* species in southern Australia

**DOI:** 10.1038/srep44787

**Published:** 2017-03-16

**Authors:** Xiaocheng Zhu, Paul A. Weston, Dominik Skoneczny, David Gopurenko, Lucie Meyer, Brendan J. Lepschi, Ragan M. Callaway, Geoff M. Gurr, Leslie A. Weston

Scientific Reports 7: Article number: 4279210.1038/srep42792; published online: 02 17 2017; updated: 03 16 2017

In the original version of this Article, the legend of Figure 4 was incorrect:

“Figure 4: There are two spelling problem in this image. Please use the revised image uploaded with this proof”.

Now reads:

“Figure 4. Distribution of haplotypes of *E. plantagineum* in southeastern Australia”.

In addition, Reference 78 was inadvertently omitted from the reference list. This reference is listed below:

Department of the Environment. Interim Biogeographic Regionalisation for Australia (Subregions) v. 7 (IBRA) [ESRI shapefile] Available from http://www.environment.gov.au/system/files/pages/5b3d2d31-2355-4b60-820c-e370572b2520/files/bioregions-new.pdf (2012).

These errors have been corrected in the HTML and PDF versions of this Article.

