# Supplementary material for: Ecology and genetics affect relative invasion success of two Echium species in southern Australia
Source: Sci Rep. 2017 Feb 17;7:42792. doi: 10.1038/srep42792 (PMC5314367; doi:10.1038/srep42792)
Supplement: Supplementary Info File [file srep42792-s1.doc]

**Ecology and genetics affect relative invasion success of two *Echium* species in southern Australia**

**Xiaocheng Zhu^1^*, Paul A. Weston^1^, Dominik Skoneczny^1^, David Gopurenko^1, 2^, Lucie Meyer^1^, Brendan J. Lepschi^3^, Ragan M. Callaway^4^, Geoff M. Gurr^1^, ^5^, Leslie A. Weston^1^**

^1^Graham Centre for Agricultural Innovation (Charles Sturt University and NSW Department of Primary Industries), Charles Sturt University, Wagga Wagga, 2678, Australia; ^2^NSW Department of Primary Industries, Wagga Wagga Agricultural Institute, Wagga Wagga, 2650, Australia; ^3^Australian National Herbarium, Centre for Australian National Biodiversity Research, Canberra, 2601, Australia; ^4^Division of Biological Sciences, University of Montana, Missoula, 59812, USA. ^5^Institute of Applied Ecology, Fujian Agriculture & Forestry University, Fuzhou 350002, China.

**Corresponding author:** Xiaocheng Zhu

Phone: +61 269334689; Fax: +61 269381861; Email: xzhu@csu.edu.au

**Supplementary Table S1.**

Pyrrolizidine alkaloids and their N-oxides detected in *Echium plantagineum* and *E. vulgare*. All compounds were found in both species, except compound (Cpd) 4, which was only found in *E. plantagineum*^31^*.*

| No. | Name |
| --- | --- |
| Cpd 1 | 3’-O-acetylechimidine-*N*-oxide |
| Cpd 2 | Lycopsamine-*N*-oxide |
| Cpd 3 | 7-O-acetyllycopsamine-*N*-oxide B |
| Cpd 4 | 7-O-acetyllycopsamine |
| Cpd 5 | 3’-O-acetylechiumine-*N*-oxide |
| Cpd 6 | Leptanthine-*N*-oxide |
| Cpd 7 | 7-angeloylretronencine-*N*-oxide |
| Cpd 8 | Echiumine-*N*-oxide B |
| Cpd 9 | Echimidine-*N*-oxide A |
| Cpd 10 | Echimidine-*N*-oxide B |
| Cpd 11 | Echiumine-*N*-oxide A |
| Cpd 12 | Echiuplatine-*N*-oxide |
| Cpd 13 | Echimiplatine-*N*-oxide |
| Cpd 14 | Uplandicine-*N*-oxide |
| Cpd 15 | Intermedine-*N*-oxide |
| Cpd 16 | 7-O-acetyllycopsamine-*N*-oxide A |
| Cpd 17 | 9-O-angelylretronencine-*N*-oxide |

**Supplementary Table S2.** Winter temperatures and summer rainfall patterns in Biogeographic region across southern Australia for the past 10 (2005-2014) and 50 (1955-2004) years.

| Biogeo-graphic region* | Mean minimum temperature of the coolest month (°C, 10 year average) | Mean minimum temperature of the coolest month (°C, 50 year average) | Mean summer rainfall (mm, 10 year average) | Mean summer rainfall (mm, 50 year average) |
| --- | --- | --- | --- | --- |
| TAS and South-eastern NSW and VIC | | | | |
| SEH | 1.20 | 0.66 | 74.63 | 67.34 |
| TSE | 2.52 | 2.37 | 44.34 | 69.83 |
| NET | 1.03 | 0.74 | 104.37 | 108.54 |
| TNS | 2.93 | 2.16 | 64.19 | 53.96 |
| BEL | 2.28 | 1.71 | 55.90 | 65.63 |
| SEC | 2.96 | 2.29 | 73.67 | 74.47 |
| NSS | 2.91 | 2.37 | 56.50 | 50.29 |
| AUA | -1.10 | -1.72 | 97.15 | 82.98 |
| NAN | 2.18 | 1.81 | 86.43 | 88.61 |
| NNC | 4.44 | 3.91 | 143.65 | 141.75 |
| SYB | 4.24 | 3.75 | 97.97 | 100.29 |
| SA and western NSW and VIC | | | | |
| SCP | 5.68 | 5.03 | 48.75 | 49.16 |
| NCP | 5.99 | 5.69 | 29.83 | 23.95 |
| RIV | 3.83 | 3.48 | 37.99 | 30.48 |
| FLB | 3.90 | 3.94 | 27.34 | 23.52 |
| DRP | 4.70 | 4.29 | 61.20 | 55.10 |
| SVP | 4.93 | 4.32 | 42.40 | 38.94 |

*Please refer to Table 1 for the code for each biogeographic region.

**Supplementary Table S3.** List of locations chosen for ecological survey.

| Species | Location* | Biogeographic region |
| --- | --- | --- |
| *Echium plantagineum* | Silverton | Broken Hill Complex |
| *Echium plantagineum* | Cobar | Cobar Peneplain |
| *Echium plantagineum* | Coombah | Murray Darling Depression |
| *Echium plantagineum* | GolGol | Murray Darling Depression |
| *Echium plantagineum* | Wagga Wagga Site 1 | NSW South Western Slopes |
| *Echium plantagineum* | Wagga Wagga Site 2 | NSW South Western Slopes |
| *Echium plantagineum* | Wagga Wagga Site 3 | NSW South Western Slopes |
| *Echium plantagineum* | Wagga Wagga Site 4 | NSW South Western Slopes |
| *Echium plantagineum* | Wagga Wagga Site 5 | NSW South Western Slopes |
| *Echium plantagineum* | Yanco | NSW South Western Slopes |
| *Echium plantagineum* | Narrandera Site 1 | NSW South Western Slopes |
| *Echium plantagineum* | Narrandera Site 2 | NSW South Western Slopes |
| *Echium plantagineum* | Adelong | NSW South Western Slopes |
| *Echium plantagineum* | Leeton | Riverina |
| *Echium plantagineum* | Hay | Riverina |
| *Echium plantagineum* | Hillston | Riverina |
| *Echium plantagineum* | Talbingo | South Eastern Highlands |
| *Echium vulgare* | Adaminaby | South Eastern Highlands |
| *Echium vulgare* | Cooma | South Eastern Highlands |
| *Echium vulgare* | Numaralla | South Eastern Highlands |
| *Echium vulgare* | Mt. Denison | South Eastern Highlands |

*Please refer to Table S4 for GPS coordinates of each location.

**Supplementary Table S4.** GPS coordinates, and types of allele(s) and haplotype assigned to samples of *E. plantagineum* (*Ep*) and *Echium vulgare* (*Ev*) collected for DNA sequencing analysis.

| Sample ID | Species | State | Location | Latitude (South) | Longitude (East) | Allele | Hap |
| --- | --- | --- | --- | --- | --- | --- | --- |
| ww14590 | *Ep* | NSW | Wagga Wagga 1 | 35.063 | 147.37 | A5 | H3 |
| ww14598 | *Ep* | NSW | Coombah | 32.983 | 141.629 | A6 | H10 |
| ww17621* | *Ev* | NSW | Michelago | 35.661 | 149.11 | A1 | H2 |
| ww17622* | *Ev* | ACT | Williamsdale | 35.58 | 149.1 | A1 | H1 |
| ww17623* | *Ev* | ACT | Bredbo | 35.911 | 149.136 | A1, A2 | H2 |
| ww17624* | *Ev* | ACT | Canberra | 35.168 | 149.154 | A1, A4 | H1 |
| ww17628 | *Ev* | NSW | Adaminaby | 35.995 | 148.791 | A1 | H1 |
| ww17629 | *Ev* | NSW | Adaminaby | 35.995 | 148.791 | A1 | H1 |
| ww17630 | *Ev* | NSW | Adaminaby | 35.995 | 148.791 | A1 | H1 |
| ww17631 | *Ev* | NSW | Adaminaby | 35.995 | 148.791 | A1 | H1 |
| ww17632 | *Ev* | NSW | Cooma | 36.14 | 149.2 | A1 | H1 |
| ww17633 | *Ev* | NSW | Cooma | 36.14 | 149.2 | A1, A2 | H1 |
| ww17634 | *Ev* | NSW | Cooma | 36.14 | 149.2 | A1, A2 | H1 |
| ww17635 | *Ev* | NSW | Cooma | 36.16 | 149.11 | A1, A3 | H1 |
| ww17636 | *Ev* | NSW | Cooma | 36.16 | 149.11 | A1, A2 | H1 |
| ww17637 | *Ev* | NSW | Cooma | 36.16 | 149.11 | A1 | H1 |
| ww17638* | *Ep* | NSW | Koorawatha | 34.053 | 148.58 | A5, A6 | H9 |
| ww17639 | *Ep* | NSW | Talbingo | 35.568 | 148.302 | A5, A6 | H5 |
| ww17640 | *Ep* | NSW | Talbingo | 35.568 | 148.302 | A5 | H5 |
| ww17641 | *Ep* | NSW | Griffith | 34.272 | 146.017 | A6 | H3 |
| ww17642 | *Ep* | NSW | Griffith | 34.272 | 146.017 | A5, A6 | H3 |
| ww17643 | *Ep* | NSW | Silverton | 31.883 | 141.228 | A5 | H9 |
| ww17644 | *Ep* | NSW | Coombah | 32.983 | 141.629 | A6 | H10 |
| ww17647 | *Ep* | NSW | Coolamon | 34.832 | 147.197 | A5, A6 | H7 |
| ww17648 | *Ep* | NSW | Coolamon | 34.832 | 147.197 | A5, A6 | H7 |
| ww17649 | *Ep* | NSW | Coolamon | 34.832 | 147.197 | A5, A6 | H7 |
| ww17650 | *Ep* | NSW | Coolamon | 34.832 | 147.197 | A6 | H7 |
| ww17651 | *Ep* | NSW | Yanco | 34.616 | 146.422 | A5, A6 | H3 |
| ww17652 | *Ep* | NSW | Yanco | 34.616 | 146.422 | A5, A6 | H3 |
| ww17653 | *Ep* | NSW | Yanco | 34.616 | 146.422 | A5, A6 | H3 |
| ww17654 | *Ep* | NSW | Yanco | 34.616 | 146.422 | A5, A6 | H3 |
| ww17655 | *Ep* | NSW | Wagga Wagga 2 | 35.052 | 147.348 | A5, A6 | H4 |
| ww17656 | *Ep* | NSW | Wagga Wagga 2 | 35.052 | 147.348 | A6 | H3 |
| ww17657 | *Ep* | NSW | Wagga Wagga 2 | 35.052 | 147.348 | A5, A6 | H4 |
| ww17658 | *Ep* | NSW | Wagga Wagga 2 | 35.052 | 147.348 | A5, A6 | H5 |
| ww17659 | *Ep* | NSW | Wagga Wagga 2 | 35.052 | 147.347 | A5, A6 | H3 |
| ww17660 | *Ep* | NSW | Wagga Wagga 2 | 35.052 | 147.347 | A5, A6 | H5 |
| ww17661 | *Ep* | NSW | Coolamon | 34.832 | 147.196 | A6 | H6 |
| ww17662 | *Ep* | NSW | Coolamon | 34.832 | 147.196 | A5, A6 | H7 |
| ww19528 | *Ep* | NSW | Wagga Wagga 3 | 35.058 | 147.352 | A5 | H5 |
| ww19529 | *Ep* | NSW | Wagga Wagga 3 | 35.058 | 147.352 | A5, A6 | H5 |
| ww19530 | *Ep* | NSW | Wagga Wagga 3 | 35.06 | 147.351 | A5, A6 | H5 |
| ww19531 | *Ep* | NSW | Wagga Wagga 3 | 35.06 | 147.351 | A5, A6 | H5 |
| ww19532 | *Ep* | NSW | Wagga Wagga 3 | 35.061 | 147.351 | A5, A6 | H5 |
| ww19533 | *Ep* | NSW | Wagga Wagga 3 | 35.061 | 147.351 | A6 | H5 |
| ww19534 | *Ep* | NSW | Wagga Wagga 3 | 35.061 | 147.351 | A5 | H5 |
| ww19535 | *Ep* | NSW | Wagga Wagga 3 | 35.061 | 147.351 | A6 | H3 |
| ww19536 | *Ep* | NSW | Wagga Wagga 3 | 35.061 | 147.351 | A5 | H5 |
| ww19537 | *Ep* | NSW | Wagga Wagga 4 | 35.043 | 147.302 | A5 | H6 |
| ww19538 | *Ep* | NSW | Wagga Wagga 4 | 35.043 | 147.302 | A5, A6 | H5 |
| ww19540 | *Ep* | NSW | Wagga Wagga 4 | 35.043 | 147.302 | A6 | H5 |
| ww19541 | *Ep* | NSW | Wagga Wagga 4 | 35.043 | 147.302 | A6 | H5 |
| ww19542 | *Ep* | NSW | Wagga Wagga 4 | 35.043 | 147.302 | A5, A6 | H5 |
| ww19544 | *Ep* | NSW | Wagga Wagga 4 | 35.043 | 147.302 | A6 | H5 |
| ww19545 | *Ep* | NSW | Wagga Wagga 4 | 35.043 | 147.302 | A5, A6 | H5 |
| ww19546 | *Ep* | NSW | Wagga Wagga 4 | 35.043 | 147.302 | A5 | H5 |
| ww19547 | *Ep* | NSW | Wagga Wagga 4 | 35.043 | 147.302 | A5, A6 | H5 |
| ww19548 | *Ep* | NSW | Wagga Wagga 3 | 35.058 | 147.352 | A5 | H5 |
| ww19549 | *Ep* | NSW | Wagga Wagga 3 | 35.058 | 147.352 | A5 | H4 |
| ww19550 | *Ep* | NSW | Wagga Wagga 3 | 35.058 | 147.352 | A5, A6 | H5 |
| ww19552 | *Ep* | NSW | Wagga Wagga 3 | 35.058 | 147.352 | A6 | H5 |
| ww19553 | *Ep* | NSW | Wagga Wagga 4 | 35.043 | 147.302 | A6 | H5 |
| ww19554 | *Ep* | NSW | Wagga Wagga 4 | 35.043 | 147.302 | A6 | H5 |
| ww19555 | *Ep* | NSW | Wagga Wagga 4 | 35.043 | 147.302 | A5, A6 | H7 |
| ww19556 | *Ep* | NSW | Wagga Wagga 4 | 35.043 | 147.302 | A5 | H7 |
| ww19557 | *Ep* | NSW | Wagga Wagga 4 | 35.043 | 147.302 | A5, A6 | H5 |
| ww19558 | *Ep* | NSW | Wagga Wagga 4 | 35.043 | 147.302 | A6 | H8 |
| ww19559 | *Ep* | NSW | Wagga Wagga 4 | 35.043 | 147.302 | A5, A6 | H8 |
| ww19560 | *Ep* | NSW | Wagga Wagga 4 | 35.043 | 147.302 | A5 | H5 |
| ww19562 | *Ep* | NSW | Wagga Wagga 4 | 35.043 | 147.302 | A5, A6 | H5 |
| ww19563 | *Ep* | NSW | Wagga Wagga 4 | 35.043 | 147.302 | A5 | H5 |
| ww19564 | *Ep* | NSW | Wagga Wagga 4 | 35.043 | 147.302 | A5, A6 | H5 |
| ww19568 | *Ep* | NSW | Narrandera 1 | 34.756 | 146.523 | A5 | H3 |
| ww19569 | *Ep* | NSW | Narrandera 2 | 34.756 | 146.523 | A5, A6 | H3 |
| ww19571 | *Ep* | NSW | Adelong | 35.296 | 148.057 | A5, A6 | H5 |
| ww19572 | *Ep* | NSW | Adelong | 35.296 | 148.057 | A5 | H5 |
| ww19573 | *Ep* | NSW | Talbingo | 35.408 | 148.291 | A5 | H5 |
| ww19574 | *Ep* | NSW | Talbingo | 35.408 | 148.291 | A5 | H8 |
| ww19575 | *Ep* | NSW | Talbingo | 35.408 | 148.291 | A5, A6 | H5 |
| ww19576 | *Ep* | NSW | Talbingo | 35.408 | 148.291 | A5, A6 | H5 |
| ww19577 | *Ep* | NSW | Talbingo | 35.408 | 148.291 | A5, A6 | H5 |
| ww19578 | *Ep* | NSW | Talbingo | 35.408 | 148.291 | A5, A6 | H7 |
| ww19579 | *Ep* | NSW | Adelong | 35.197 | 147.885 | A5, A6 | H5 |
| ww19580 | *Ep* | NSW | Adelong | 35.197 | 147.885 | A5, A6 | H5 |
| ww19581 | *Ep* | NSW | Adelong | 35.197 | 147.885 | A6 | H5 |
| ww19582 | *Ev* | NSW | Numeralla | 36.173 | 149.349 | A1, A2 | H1 |
| ww19583 | *Ev* | NSW | Numeralla | 36.173 | 149.349 | A2 | H2 |
| ww19584 | *Ev* | NSW | Cooma | 36.246 | 149.027 | A2 | H1 |
| ww19585 | *Ev* | NSW | Cooma | 36.246 | 149.027 | A1, A3 | H1 |
| ww19586 | *Ev* | NSW | Adaminaby | 35.935 | 148.663 | A2 | H1 |
| ww19587 | *Ev* | NSW | Adaminaby | 35.935 | 148.663 | A1, A2 | H1 |
| ww19588 | *Ev* | NSW | Talbingo | 35.893 | 148.518 | A1, A2 | H2 |
| ww19589 | *Ev* | NSW | Talbingo | 35.893 | 148.518 | A1, A2 | H2 |
| ww19590 | *Ep* | NSW | Wagga Wagga 5 | 35.136 | 147.385 | A5, A6 | H7 |
| ww19591 | *Ep* | NSW | Wagga Wagga 6 | 35.113 | 147.305 | A5 | H7 |
| ww19592 | *Ep* | NSW | Leeton | 34.533 | 146.409 | A6 | H5 |
| ww19593 | *Ep* | NSW | Leeton | 34.533 | 146.409 | A6 | H4 |
| ww19594 | *Ep* | NSW | Yanco | 34.706 | 146.508 | A6 | H5 |
| ww19595 | *Ep* | NSW | Yanco | 34.706 | 146.508 | A6 | H5 |
| ww19596 | *Ep* | NSW | Hay | 34.497 | 144.831 | A6 | H3 |
| ww19597 | *Ep* | NSW | Hay | 34.497 | 144.831 | A5, A6 | H11 |
| ww19598 | *Ep* | NSW | Mildura | 34.02 | 141.847 | A5, A6 | H3 |
| ww19599 | *Ep* | NSW | Mildura | 34.02 | 141.847 | A5, A6 | H12 |
| ww19600 | *Ep* | NSW | Coombah | 32.982 | 141.628 | A5 | H5 |
| ww19601 | *Ep* | NSW | Coombah | 32.982 | 141.628 | A5, A6 | H4 |
| ww19602 | *Ep* | NSW | Gol Gol | 34.195 | 142.239 | A6 | H10 |
| ww19603 | *Ep* | NSW | Gol Gol | 34.195 | 142.239 | A6 | H10 |
| ww19604 | *Ep* | NSW | Silverton | 31.883 | 141.216 | A5, A6 | H9 |
| ww19605 | *Ep* | NSW | Silverton | 31.883 | 141.216 | A5 | H11 |
| ww19606 | *Ep* | TAS | Launceston | 41.452 | 147.172 | A6 | H10 |
| ww19607 | *Ep* | TAS | Kingston | 42.998 | 147.319 | A5, A6 | H13 |
| ww19608 | *Ep* | TAS | St Helens | 41.319 | 148.21 | A6 | H5 |
| ww19609 | *Ep* | NSW | Griffith | 34.244 | 145.976 | A5, A6 | H4 |
| ww19610 | *Ep* | NSW | Griffith | 34.244 | 145.976 | A6 | H5 |
| ww19611 | *Ep* | NSW | Hillston | 32.992 | 145.9 | A6 | H7 |
| ww19612 | *Ep* | NSW | Hillston | 32.992 | 145.9 | A6 | H5 |
| ww19613 | *Ep* | NSW | Cobar | 31.51 | 145.744 | A6 | H5 |
| ww19614 | *Ep* | NSW | Cobar | 31.51 | 145.744 | A5, A6 | H5 |
| ww19615 | *Ep* | NSW | White Cliffs | 31.018 | 143.045 | A5, A6 | H10 |
| ww19616 | *Ep* | NSW | White Cliffs | 31.018 | 143.045 | A5, A6 | H3 |
| ww19617 | *Ep* | NSW | White Cliffs | 30.852 | 143.087 | A5, A6 | H3 |
| ww19618 | *Ep* | NSW | White Cliffs | 30.852 | 143.087 | A5 | H3 |
| ww19619 | *Ep* | NSW | White Cliffs | 30.85 | 143.089 | A5, A6 | H3 |
| ww19620 | *Ep* | NSW | Cobar | 31.578 | 145.136 | A6 | H4 |
| ww19622* | *Ep* | SA | St Agnes | 34.85 | 138.68 | A6 | H11 |
| ww19623* | *Ep* | SA | Mintaro | 33.91 | 138.71 | A5 | H10 |
| ww19626 | *Ep* | NSW | Coombah | 32.982 | 141.628 | A5 | H10 |
| ww19627* | *Ep* | WA | Dongara | 29.41 | 115.06 | A5 | H10 |
| ww19629* | *Ep* | WA | NA | NA | NA | A5, A6 | H8 |
| ww19631* | *Ep* | WA | Narrogin | 32.926 | 117.163 | A5, A6 | H14 |
| ww19632* | *Ep* | WA | Northam | 31.65 | 116.66 | A6 | H15 |
| ww19635* | *Ep* | VIC | Mulgrave | 37.921 | 145.191 | A6 | H5 |
| ww19636* | *Ep* | VIC | Barmah | 35.91 | 144.96 | A5, A6 | H3 |
| ww19637* | *Ep* | NT | Alice Springs | 23.2 | 133.75 | A6 | H3 |
| ww19638* | *Ep* | ACT | Canberra | 35.26 | 149.1 | A5, A6 | H3 |
| ww19639* | *Ep* | ACT | Canberra | 35.45 | 148.91 | A6 | H4 |
| ww19640* | *Ep* | ACT | Canberra | 35.41 | 149.08 | A5 | H8 |
| ww19641* | *Ep* | QLD | Goondiwindi | 28.52 | 150.271 | A6 | H10 |
| ww19642* | *Ep* | QLD | Warwick | 28.224 | 152.03 | A6 | H5 |
| ww19643* | *Ep* | QLD | Smithlea | 28.837 | 151.071 | A6 | H4 |
| ww19644* | *Ep* | NSW | Scotia | 33.272 | 141.212 | A5 | H13 |
| ww19645* | *Ep* | NSW | Crowthe | 34.159 | 148.626 | A5 | H5 |
| ww19646* | *Ep* | NSW | Tundulya | 30.85 | 145.26 | A6 | H10 |
| ww19647* | *Ep* | NSW | Tumut | 35.33 | 148.33 | A5, A6 | H8 |
| ww19649* | *Ep* | NSW | Hobbys Yards | 33.585 | 149.268 | A6 | H3 |
| ww20699 | *Ep* | NSW | Bathurst | 33.463 | 149.476 | A6 | H3 |
| ww20700 | *Ep* | NSW | Bathurst | 33.463 | 149.476 | A6 | H3 |
| ww21241 | *Ev* | NSW | Bathurst | 33.463 | 149.476 | A1 | H1 |
| ww21242 | *Ev* | NSW | Bathurst | 33.463 | 149.476 | A1 | H1 |
| ww21244 | *Ev* | NSW | Adaminaby | 35.995 | 148.791 | A1 | H2 |
| ww21245 | *Ep* | VIC | Bandiana | 36.153 | 146.88 | A5, A6 | H5 |
| ww21246 | *Ep* | VIC | Bandiana | 36.153 | 146.88 | A5, A6 | H5 |
| ww21247 | *Ep* | VIC | Kangaroo Flat | 36.853 | 144.195 | A5 | H5 |
| ww21248 | *Ep* | VIC | Kangaroo Flat | 36.853 | 144.195 | A6 | H5 |

* indicates samples were donated by Australian National Herbarium.


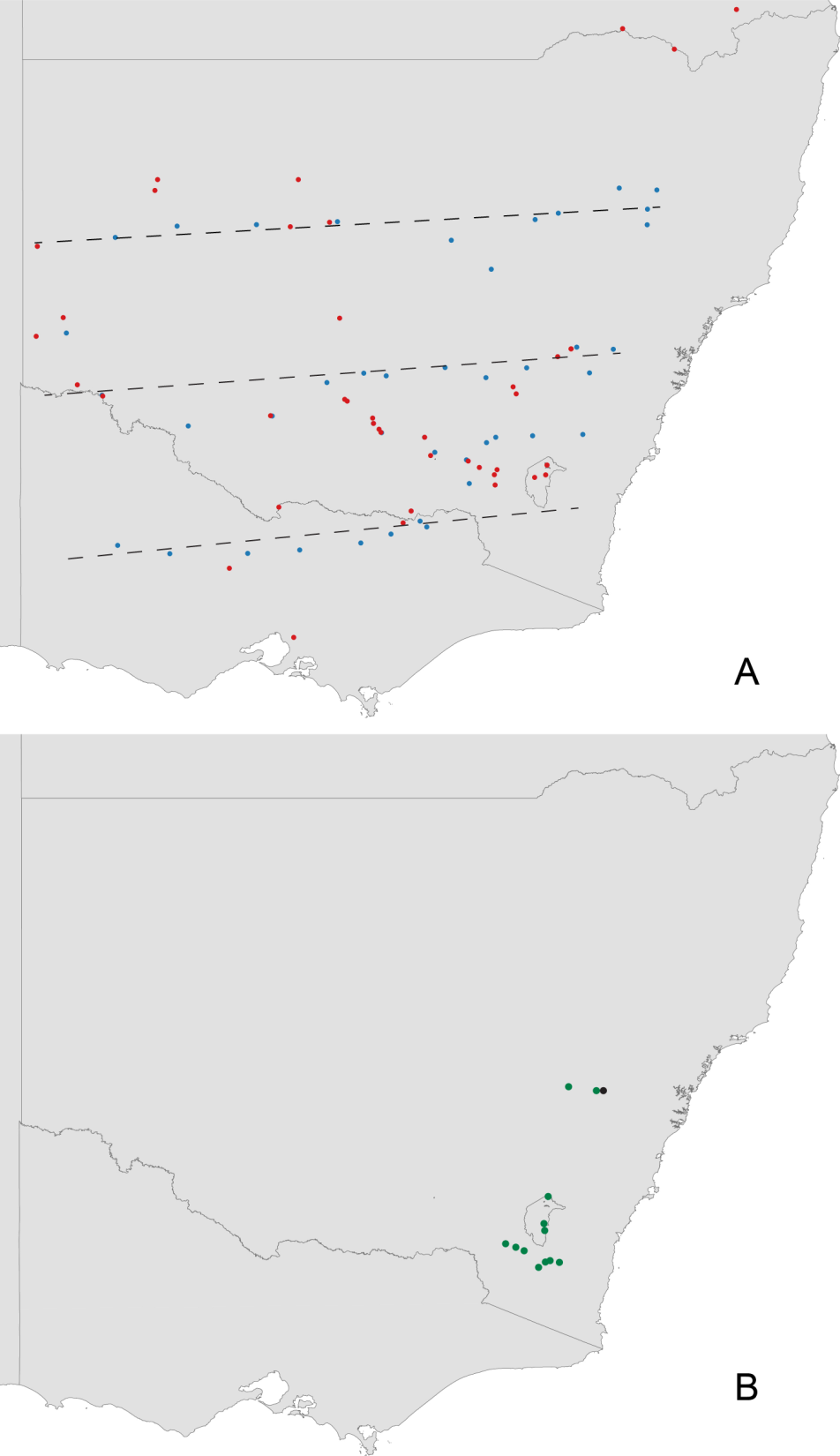


**Supplementary Figure S1.** Survey of distribution of *E. plantagineum* (A) and *E. vulgare* (B) in south-eastern Australia ^33^ using three longitudinal transects (dashed line) plus additional sampling conducted in the Riverina NSW. Red and green circles indicate the locations where *E. plantagineum* and *E. vulgare* were collected for further genetic analyses, respectively. This map is a derivative of “State and Territory ASGC Ed 2011 Digital Boundaries in ESRI Shapefile Format” sourced from the Australian Bureau of Statistics, used under CC BY 2.5 (<https://creativecommons.org/licenses/by/2.5/au/>) and modified using ArcGIS 10.3.1 software by Esri ([http://www.esri.com](http://www.esri.com/)).


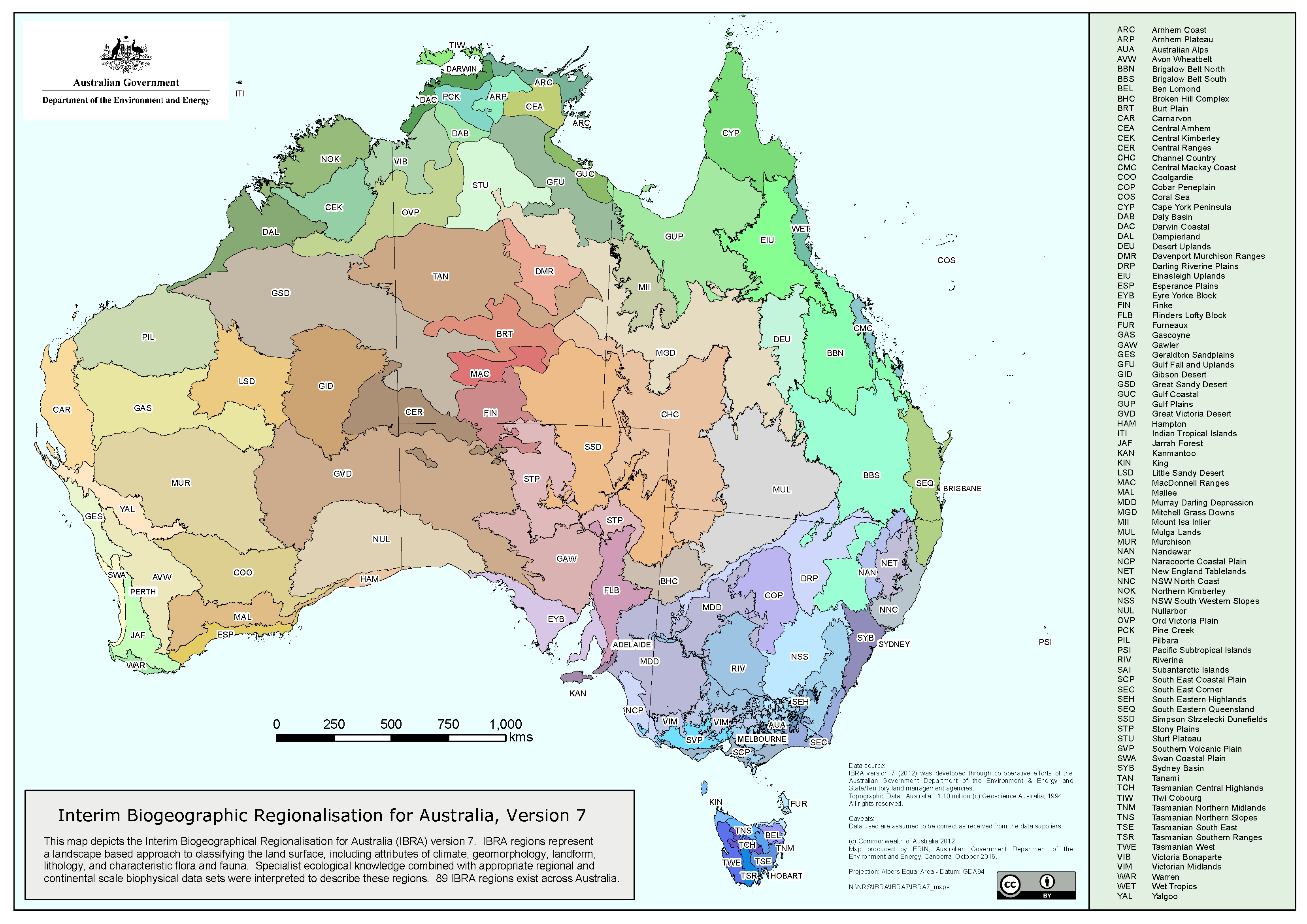


**Supplementary Figure S2.** “Interim Biogeographic Regionalisation for Australia, Version 7” by © Commonwealth of Australia 2016, used under [CC BY 4.0](https://creativecommons.org/licenses/by/4.0/) (<https://creativecommons.org/licenses/by/4.0/>), showing 89 biogeographic regions of Australia divided according to the common climate, geology, landform, native vegetation and species information^78^. Map downloaded from <http://www.environment.gov.au/system/files/pages/5b3d2d31-2355-4b60-820c-e370572b2520/files/bioregions-new.pdf>.


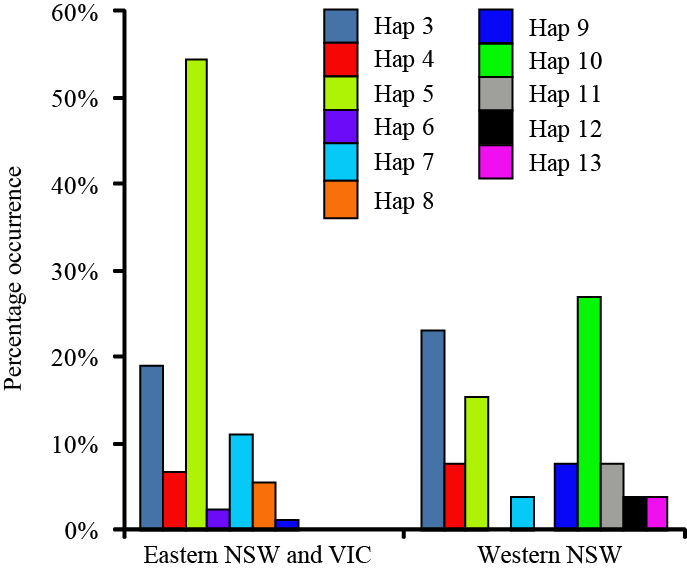


**Supplementary Figure S3.** Percentage of occurrence of various haplotypes of *E. plantagineum* in NSW and eastern VIC. Haplotypes 1 and 2 are haplotypes of *E. vulgare* and are not presented.


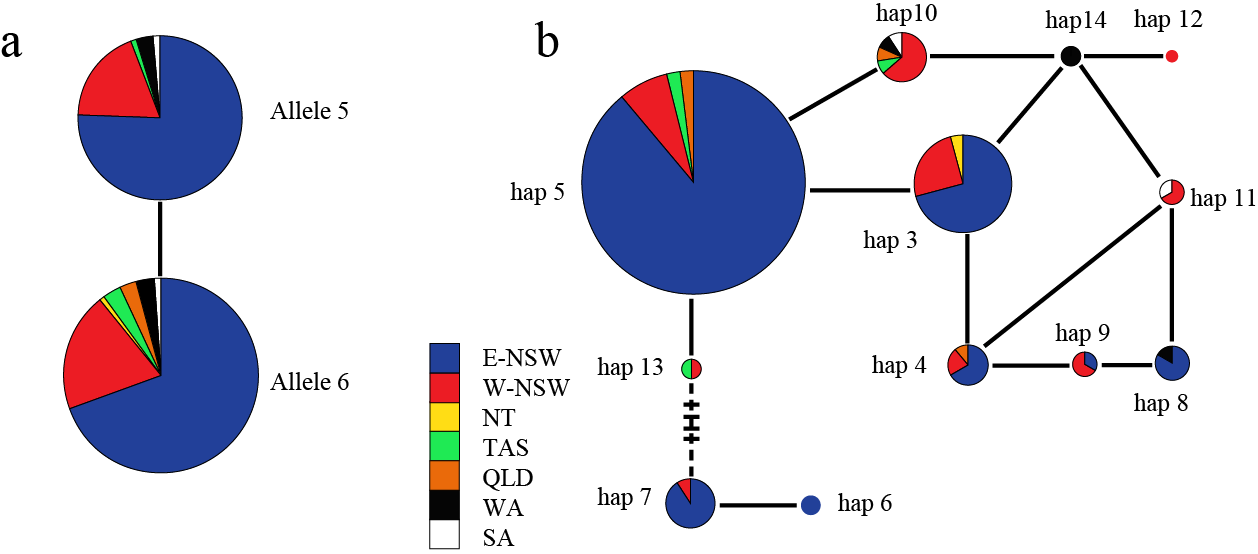


**Supplementary Figure S4.** Nuclear (a) and chloroplast (b) 95% parsimony networks of *E. plantagineum* collected across Australia. Alleles 5 and 6 represent two nuclear alleles while Hap 3-14 represent 12 haplotypes associated with the chloroplast genome. E-NSW: eastern New South Wales (NSW) and Victoria; W-NSW: western NSW; NT: Northern Territory; TAS: Tasmania; QLD: Queensland; WA: Western Australia; SA: South Australia.
